# Designing an experimental HIV/HCV intervention to promote the safe re-use of drug preparation materials by injection drug users in Puerto Rico

**DOI:** 10.1186/1477-7517-5-14

**Published:** 2008-04-28

**Authors:** H Ann Finlinson, Héctor M Colón, Juan Negrón, Rafaela R Robles

**Affiliations:** 1 Center for Addiction Studies, School of Medicine, Universidad Central del Caribe, PO Box 60327, Bayamón, 00960-6032, Puerto Rico

## Abstract

Injection drug users (IDUs) in San Juan, Puerto Rico are characterized by high rates of daily injecting, injection of shared drugs, re-use of injection syringes, and use of shooting galleries. They lack adequate access to new injection syringes and drug preparation equipment, and experience elevated rates of HIV and HCV infection. Between April and August, 2006, researchers and active IDUs collaborated in the development of an experimental HIV/HCV intervention aimed at identifying drug preparation items and practices that will enable IDUs to make drug solutions without potentially contaminated injection syringes contacting materials used to prepare drugs. The collaboration involved discussing and testing a variety of drug preparation items and practices in office and community settings. The process was repeated until concerns that had been raised were resolved, and a tentative set of intervention items and practices to be evaluated in a community field trial was identified. Throughout, a strong emphasis was placed on the capacity of an item or practice to address common problems confronted by IDUs (blunted needles, clogged syringes, injected particles) in addition to the core aim of reducing contamination of preparation materials by blood in injection syringes.

This report describes the final selection of items and practices: 1) A small water bottle that permits IDUs to add approximately .05 cc water drops directly to drug powder in cookers; 2) A preparation syringe (a type of ancillary equipment not used for injecting) that permits IDUs to pull up a measurable amount of water to add to drug powder, an alternative to producing water drops; 3) A filtering device, the Sterifilt filter, attached to a preparation syringe, which eliminates the need for cotton or cigarette filters; 4) Use of a preparation syringe to distribute drug solution by backloading to injection syringe(s); 5) A small water bottle enabling IDUs to clean injection syringes by backload rinsing. The overarching aim of this experimental HIV/HCV intervention was to promote the safe re-use of drug preparation and injection items, and to impact the large number of IDUs in San Juan who maintain personal injection syringes, but currently use communal ancillary equipment in shooting galleries and inject drug solutions prepared with other IDUs' injection syringes.

## Background

HIV and HCV infection among injection drug users (IDUs) in Puerto Rico constitutes a public health emergency even after two decades of epidemiological and anthropological research [1]. Recent studies of street-recruited IDUs in San Juan document HIV seroprevalence of 21% [2] and HCV seroprevalence of 89% [3]. Behind these disturbing statistics are consistently high levels of risky injection behaviors, including number of daily injections [4], utilization of shooting galleries [5], joint drug purchase and injection of shared drugs [6], and re-use of injection equipment [7]. At present, IDUs are unable to access adequate numbers of new syringes or ancillary injection equipment. The estimated 15,935 IDUs residing in San Juan [8] inject a mean of 184 times each month [9], meaning that 2,932,040 new syringes need to be circulated monthly if injection syringes are not to be re-used. The one source of free syringes and ancillary equipment is the needle exchange program which lacks funding to routinely provide HIV/HCV prevention kits [10] and currently distributes less than 1% of the syringes needed monthly (J. Negron, unpublished data). Not surprisingly, IDUs attempt to extend the life of each syringe, resulting in a mean reuse rate of six times per syringe [11].

In response to this public health crisis, researchers and IDUs from San Juan are collaborating in an experimental HIV/HCV prevention intervention aimed at identifying drug preparation items and practices that will enable IDUs to make a drug solution using ancillary equipment not exposed to blood, and to transfer the solution to the barrel of an injection syringe after its plunger is removed (i.e., by backloading). This report describes the initial phase in the development of the intervention in which potential items and practices were identified and tested through an iterative process until concerns raised in office and community settings were resolved. Throughout, the capacity of an item or practice to address common problems IDUs confront (e.g., blunted needles, injected particles) received special attention, in addition to the central aim of reducing contamination of preparation materials by blood in injection syringes.

## Methods

Selection criteria for participating IDUs included: current drug injecting, age 18 or older, Puerto Rican self-identification, residential stability, and expressed interest in drug injecting innovation. An outreach worker contacted 18 potential participants; 10 (7 males, 3 females) were selected after a screening interview with the project ethnographer. Five IDUs were recruited from one San Juan community and five from a second community. Participants received information about study objectives, activities, confidentiality protections and compensation. Participants received $20.00 for each of three office-based sessions; 9 of the 10 participants attended all three sessions. The amount of compensation was comparable to payments for focus group participation in recent HIV/HCV studies. Participants were not compensated monetarily for testing potential intervention items in community settings. The UCC Institutional Review Board approved all study procedures.

The office-based sessions were held in community centers. Three rounds of sessions were conducted between April and August, 2006. For each round, a session was held in one community, and on the following day a session was held in the other community. Thus the three rounds produced a total of six sessions. At the beginning of each session, participants were offered refreshments; sessions lasted about 1.5 hours.

The content of office-based sessions changed as the intervention evolved, however all sessions followed a similar structure: introductory comments about the study's objectives; demonstrations and discussions (e.g., current drug preparation practices, potential intervention items and practices); concluding remarks about arrangements for supplying participants with samples of items under consideration.

Audio-visual recordings were made of each session with the participants' prior consent. In Round I, a single stationary camera recorded the facilitator and session participants. In Rounds II and III, a second hand-held camera was added, which provided close-ups of participants' hands as they demonstrated or practiced using different items. Within two days of a round of sessions, the research team examined audio-visual recordings, taking detailed notes and transcribing participants' comments. The research team then discussed the issues raised during the sessions and identified steps to be taken for up-coming sessions.

## Findings

Researchers and IDUs discussed and tested several drug preparation items and practices before making a final selection, described below, to be evaluated in a community field trial.

### Forming the drug solution (putting water into a cooker containing drug powder)

**Figure 1:**
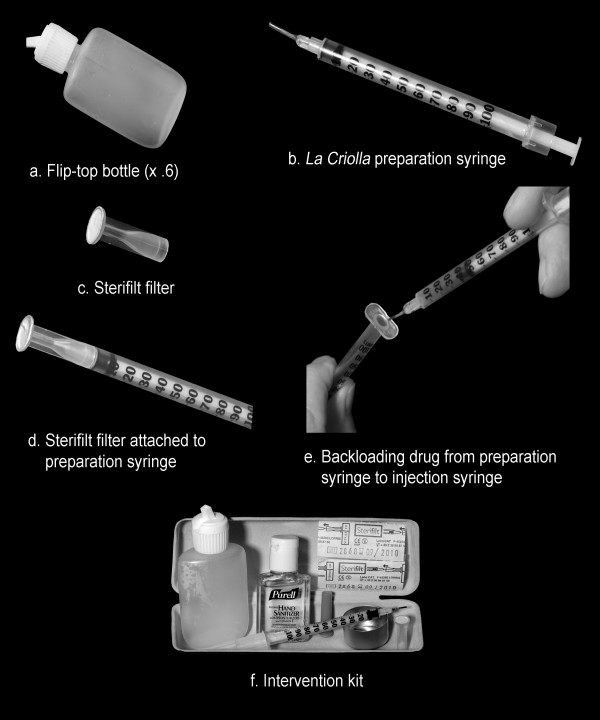
**Final selection of intervention items**. A small flip-top water bottle (1a) permits IDUs to add approximately .05 cc water drops directly to drug powder in cookers and to clean injection syringes by backload rinsing. A preparation syringe (1b), a type of ancillary equipment not used for injecting, permits IDUs to pull up a measurable amount of water to add to drug powder, an alternative to producing water drops with the small water bottle. The Sterifilt filter (1c) attached to a preparation syringe (1d) eliminates the need for cotton or cigarette filters. Drug solution, having been filtered and drawn up into the preparation syringe, is transferred to an injection syringe (1e). The intervention kit (1f) includes a flip-top water bottle, cooker, preparation syringe (*La Criolla*), Sterifilt filters, and bottle of alcohol sanitizer.

Two potential intervention items and practices were identified. (1) The flip-top bottle (Figure 1a) is easily refilled and permits IDUs to add approximately .05 cc water drops to powder in cookers. This particular unit of water facilitates quick calculation of the total amount of water needed (e.g., 8 drops for .40 cc water). The bottle is a 1.25 oz natural color, low-density polyethylene (LDPE) oval bottle with 18 mm flip top cap and 4 mm uncontrolled dropper orifice. The unit cost of this item is $0.23. (2) A preparation syringe (i.e., a syringe that is not used for injecting and is considered a type of ancillary equipment) referred to as *La Criolla *(Figure 1b) permits IDUs to pull up a measurable amount of water to add to drug powder, an alternative to producing water drops using the flip-top bottle. This preparation syringe is a modified 1 cc 28 gauge insulin syringe commonly used by IDUs in San Juan. The metal needle is replaced with a 3/8 inch segment of Teflon tubing affixed to the syringe with epoxy, and a thin layer of clear lacquer is applied to the numbers on the barrel. *La Criolla *costs $0.27 each to produce and has the advantages of number markings familiar to IDUs and number markings that do not rub off easily.

### Filtering the drug solution

One filtering device, the Sterifilt filter (Figure 1c), which fits on the preparation syringe described above (Figure 1d), was selected after tests conducted by researchers and IDUs. The Sterifilt filter is a commercially available harm reduction device consisting of a polypropylene body and paper membrane; it is designed to fit onto a syringe and be used a single time. This filter costs $0.21 per unit and blocks "90% of particles measuring less than five microns," and retains three times less drug than filters commonly used by IDUs, including cotton and cigarette filters [12]. Of all items considered during the study, IDUs expressed greatest interest in the Sterifilt filter primarily because it blocks small particles that clog syringes and, if injected, cause a painful reaction known as "cotton fever." Also, filtering solutions with a Sterifilt filter attached to a preparation syringe result in less use, and thus less blunting, of injection syringe needles.

Study researchers conducted ten office-based trials to determine the number of possible filter re-uses. Each trial, repeated 23–29 times, involved drawing up approximately .5 cc water with a Sterifilt-fitted syringe, removing the filter, holding the membrane against a light bulb, and visually inspecting for ruptures and separations of the paper membrane from the polypropylene body. Survival probability was calculated after each trial; the filter had a 90% probability of surviving six uses.

### Distributing filtered drug solution to injection syringe(s)

Researchers and IDUs identified a potential intervention practice of using the preparation syringe, *La Criolla*, to distribute filtered drug solution by backloading to injection syringes (Figure 1e). Advantages include: imperceptible drug retention (i.e., low dead space), widespread familiarity with backloading, familiar number markings on preparation syringe that facilitate determination of solution volume and fair division of portions, and a Teflon needle structure that permits safe transfer of drug solution to injection syringe(s).

### Cleaning injection syringe

IDUs in San Juan normally rinse a re-used injection syringe by drawing up and expelling a small amount of water (e.g., .40 cc) through the syringe's metal needle. An alternative to this practice, referred to as backload rinsing, was identified. It involves removing the plunger from the injection syringe; directing a strong, narrow stream of water from the flip-top bottle (Figure 1a) into the syringe barrel, filling its entire length; replacing the plunger; expelling the water out the metal needle. Advantages include: a) the upper portion of the barrel is rinsed (this part is often exposed to drug solution but inadequately rinsed by the traditional cleaning method); b) the need for small, communal water receptacles (*tapitas*) for rinsing injection syringes is eliminated; c) IDUs gain personal control over the water used to rinse injection syringes.

## Discussion

IDUs in San Juan are unable to access adequate numbers of new syringes and drug preparation equipment, a situation unlikely to change in the foreseeable future. This report describes initial steps in the development of an experimental intervention that enables IDUs to prepare drug solutions without HIV/HCV-contaminated injection syringes contacting drug preparation materials. The overarching aim is to promote the safe re-use of drug preparation and injection equipment, and to impact the large number of IDUs in San Juan who maintain personal injection syringes but use communal ancillary equipment in shooting galleries and inject drug solutions prepared with other IDUs' injection syringes.

The high level of participant attendance at the three sessions has been critical to the realization of study goals, and was achieved by: a) offering substantial monetary compensation; b) holding small group sessions to facilitate detailed demonstrations and discussions of injection practices; c) selecting participants on the basis of neighborhood residence to ensure social ease at the sessions; d) conducting sessions at community centers close to where participants live and engage in daily activities. An additional distinctive factor in sustaining participant interest was the overt recognition by researchers of the expert knowledge and skills possessed by participating IDUs.

Both male and female IDUs contributed to this study but gender did not appear to be relevant in the discussions, testing, or selection of items for the intervention kit (Figure 1f). All of the participants were veteran IDUs with generally comparable drug-related knowledge and skills, and access to monetary resources. They engaged in similar drug injection practices, each maintaining a personal syringe but engaging in indirect sharing (e.g., using communal cookers and water receptacles, distributing the contents of their syringe to others, injecting drug solutions prepared by other IDUs). Even though gender was not a significant factor in this initial study, it is possible that important gender differences may exist in the ability of IDUs to carry or store intervention items because kits are easily added to women's purses, and women are less subject than males to police searches in San Juan. Gender differences in adoption rates may also appear if the intervention is introduced to a larger sample of IDUs in which there are differences between males and females in injection practices (e.g., women in certain circumstance may be less likely to control the drug preparation process). These issues will be explored when the intervention is evaluated in community trials. It is hoped that this intervention proves to be beneficial for IDUs in Puerto Rico and perhaps for other IDU populations that experience inadequate access to programs, including syringe exchange programs, shown to be effective in addressing HIV and viral hepatitis.

## Authors' contributions

HAF contributed to study conception and design, and data analysis and interpretation, and drafted the manuscript. HMC contributed to study conception and design, oversaw data collection, contributed to data analysis and interpretation, and helped draft the manuscript. JN participated in data collection and interpretation. RRR advised on study design and data interpretation.
